# Supplementary material for: Virological investigation and comparative genomic analysis of elephant endotheliotropic herpesvirus 1B infection in an Australian captive herd of Asian elephants (Elephas maximus)
Source: PLoS One. 2026 May 29;21(5):e0345964. doi: 10.1371/journal.pone.0345964 (PMC13221008; doi:10.1371/journal.pone.0345964)
Supplement: Attachment — Submitted filename: Review EEHV1B.pdf [file pone.0345964.s005.pdf]

Virological investigation of elephant endotheliotropic herpesvirus 1B infection in an Australian captive herd of Asian elephants

The manuscript provides valuable information on the first reported case of EEHV1B infection in zoo Asian elephants in Australia, with important genetic insights that will interest the readership. However, several points require clarification or expansion to strengthen the manuscript. Please address the following comments to improve clarity, accuracy, and completeness.

Title: As the manuscript primarily focuses on genetic characterization, I encourage revising the title to more clearly reflect the genetic or whole-genome analysis aspect of this first EEHV1B case in Australia.

General Comments: The manuscript inconsistently uses multiple identifiers for the same individual/sample (e.g., *Elephant 7*, *AUP_01*, *EEHV1B_AUP_01_2023*). Please standardize terminology throughout to avoid confusion.

Materials and Methods

Line 152 – Sample collection

Please clarify the sampling frequency of trunk washes. Were samples collected every two weeks, monthly, or only opportunistically? The current description is unclear.

Line 172 – Preservation methods

Because the study compares different preservation types, please provide more detail on the storage protocol, including:

- Approximate tissue size or weight placed in each preservative
- Volume (mL) of preservative used

This information is important for interpreting differences between preservation methods.

Line 196 – qPCR platforms

Different qPCR machines were used for tissue versus trunk wash/whole blood samples. Please explain the rationale and would this effect the interpretation?

Results

Lines 321–336 – Clinical progression

- Please include the treatment protocol and platelet levels during Days 0–3, as these provide essential context for interpreting clinical progression alongside viral load dynamics.
- Line 329 reports *100,000 VGE/mL* on Day 0, whereas Line 334 reports *4,210,000 VGE/mL*. Please verify which value is correct.

Lines 337–364 – Trunk wash results
It is unclear whether trunk wash positives represent EEHV1B specifically or EEHV1 more generally. For example, Line 343 refers to an EEHV1B spike in Elephant 2, but Figure 1 labels results as EEHV1. Please clarify:

- Which results distinguish EEHV1A vs EEHV1B
- Which samples were typed as EEHV1B
- Ensure consistent terminology in both text and figures.

Figure 1
The x-axis labels are not clear. Please revise to improve readability and interpretation.

Discussion

In addition to including treatment details in the Results, please expand the Discussion to address treatment outcome, including factors contributing to treatment success or failure.

Additional point regarding preservation performance:
The manuscript does not discuss why RNAlater appeared ineffective for most tissue samples. Please expand on potential explanations.

Additionally, for samples preserved under different conditions—including non-preserved tissues—please discuss why some yielded “N/A” results. In theory, non-preserved samples should still contain detectable host DNA. Please consider whether these outcomes may reflect:

- Variation in DNA extraction quality
- Tissue degradation
- Differences in preservation performance
- Personal error

A brief discussion of these possibilities will help readers interpret the preservation comparison.
