# Supplementary material for: Virological investigation and comparative genomic analysis of elephant endotheliotropic herpesvirus 1B infection in an Australian captive herd of Asian elephants (Elephas maximus)
Source: PLoS One. 2026 May 29;21(5):e0345964. doi: 10.1371/journal.pone.0345964 (PMC13221008; doi:10.1371/journal.pone.0345964)
Supplement: Attachment — Submitted filename: Response to reviewers.docx [file pone.0345964.s006.docx]

**Response to reviewers**

**Virological investigation of elephant endotheliotropic herpesvirus 1B infection in an Australian captive herd of Asian elephants (*Elephas maximus)***

Dear editor/s and reviewers,

Thank you for your positive feedback and suggestions. Please find individual responses to each revision suggestion outlined below. Responses are indicated by blue text. Line numbers (**L xx**) for reference are those listed in the marked-up document.

**Editor:**

When submitting your revision, we need you to address these additional requirements.
1. Please ensure that your manuscript meets PLOS ONE's style requirements, including those for file naming. The PLOS ONE style templates can be found at
[https://journals.plos.org/plosone/s/file?id=wjVg/PLOSOne_formatting_sample_main_body.pdf](https://track.editorialmanager.com/CL0/https:%2F%2Fjournals.plos.org%2Fplosone%2Fs%2Ffile%3Fid=wjVg%2FPLOSOne_formatting_sample_main_body.pdf/1/010f019d569f021e-f57d9b13-73e7-4c8d-a92a-0739a0f00a77-000000/bluYD-oNIo-8X2jqoCqwQZQt8KhuiVOa2Jh_zQT3TLc=257) and [https://journals.plos.org/plosone/s/file?id=ba62/PLOSOne_formatting_sample_title_authors_affiliations.pdf](https://track.editorialmanager.com/CL0/https:%2F%2Fjournals.plos.org%2Fplosone%2Fs%2Ffile%3Fid=ba62%2FPLOSOne_formatting_sample_title_authors_affiliations.pdf/1/010f019d569f021e-f57d9b13-73e7-4c8d-a92a-0739a0f00a77-000000/lgCrYxrkajFvmJ5TYJYns1Fpig7MHcRW_Q5kD6WQoJE=257)

Response: The document has been formatted to the style guide, including the file naming convention.

2. To comply with PLOS One submissions requirements, in your Methods section, please provide additional information regarding the experiments involving animals and ensure you have included details on (1) methods of sacrifice, (2) methods of anesthesia and/or analgesia, and (3) efforts to alleviate suffering.

Response:

The study primarily encompasses post-mortem analysis of a clinical case of EEHV. All samples collected and treatments administered were for the purposes of clinical management of the case, independent of this report. No animal experimentation occurred within this study.

3. Please provide a complete Data Availability Statement in the submission form, ensuring you include all necessary access information or a reason for why you are unable to make your data freely accessible. If your research concerns only data provided within your submission, please write "All data are in the manuscript and/or supporting information files" as your Data Availability Statement.

Response: A revised data availability statement is now provided as suggested: “All data are in the manuscript and/or supporting information files”.

4. When completing the data availability statement of the submission form, you indicated that you will make your data available on acceptance. We strongly recommend all authors decide on a data sharing plan before acceptance, as the process can be lengthy and hold up publication timelines. Please note that, though access restrictions are acceptable now, your entire data will need to be made freely accessible if your manuscript is accepted for publication. This policy applies to all data except where public deposition would breach compliance with the protocol approved by your research ethics board. If you are unable to adhere to our open data policy, please kindly revise your statement to explain your reasoning and we will seek the editor's input on an exemption. Please be assured that, once you have provided your new statement, the assessment of your exemption will not hold up the peer review process.

5. We note that the grant information you provided in the ‘Funding Information’ and ‘Financial Disclosure’ sections do not match. When you resubmit, please ensure that you provide the correct grant numbers for the awards you received for your study in the ‘Funding Information’ section.

Response: Apologies for any incongruence in information listed. The detail listed below in the funding statement is correct:

“This study was funded by generous donation from the Cybec Foundation to the University of Melbourne (J.D., J.W.) (<https://www.cybec.org/>), by the Australian Research Training Program Scholarship (Stipend) (J.W.), Australian Government (doi.org/10.82133/C42F-K220), and by the Ernie Stewart Memorial Scholarship (J.W.), University of Melbourne (<https://scholarships.unimelb.edu.au/awards/ernie-stewart-memorial-scholarship>). Funding sources were not involved in study design, collection, analysis or interpretation of data, nor writing of the report.”

Upon resubmission, we will ensure this reflected in both the financial disclosure and funding information sections on resubmission. We do not have grant numbers available as the Cybec funding was a philanthropic gift bestowal to the University and the other two funding sources are scholarships.

6. Thank you for stating the following in the Acknowledgments Section of your manuscript:
This project was funded by Cybec Foundation, the Australian Research Training Program Grant (JW) and the Ernie Stewart Memorial Grant (JW). The authors acknowledge the veterinary department and the elephant keeper team for their dedicated efforts to the treatment of this elephant and the collection of diagnostic samples, as well as the Melbourne Veterinary School Anatomic Pathology team for provision of tissue samples.

We note that you have provided funding information that is not currently declared in your Funding Statement. However, funding information should not appear in the Acknowledgments section or other areas of your manuscript. We will only publish funding information present in the Funding Statement section of the online submission form. Please remove any funding-related text from the manuscript and let us know how you would like to update your Funding Statement. Currently, your Funding Statement reads as follows:
This study was funded by generous donation from the Cybec Foundation to the University of Melbourne (J.D., J.W.) (<https://www.cybec.org/>), by the Australian Research Training Program Scholarship (Stipend) (J.W.), Australian Government (doi.org/10.82133/C42F-K220), and by the Ernie Stewart Memorial Scholarship (J.W.), University of Melbourne (<https://scholarships.unimelb.edu.au/awards/ernie-stewart-memorial-scholarship>). Funding sources were not involved in study design, collection, analysis or interpretation of data, nor writing of the report.

Please include your amended statements within your cover letter; we will change the online submission form on your behalf.

Response: We have revised our acknowledgements (**L792**) to only identify non-financial acknowledgements, including the work of the vets and pathology teams involved in the case. All funding related text has been removed from acknowledgements.

7. Please amend your authorship list in your manuscript file to include author Jack William Wheelahan.

8. Please amend the manuscript submission data (via Edit Submission) to include author Jack W. Wheelahan.

Response: There appears to be a mismatch between the manuscript and submission authorship list, by inclusion of my full middle name. To clarify, they refer to the same individual, and I will edit the submission data to reflect the manuscript listed author to Jack W. Wheelahan.

9. Please amend your list of authors on the manuscript to ensure that each author is linked to an affiliation. Authors’ affiliations should reflect the institution where the work was done (if authors moved subsequently, you can also list the new affiliation stating “current affiliation:….” as necessary).

Response: I have reviewed this and I believe all authors had affiliations listed on the first submission, I cannot identify any errors compared with the submission guidelines. One co-author has requested I add an additional current affiliation. Please indicate if there are any issues requiring further attention.

10. We note that there is identifying data in the Supporting Information file <S1_file.xlsx>. Due to the inclusion of these potentially identifying data, we have removed this file from your file inventory. Prior to sharing human research participant data, authors should consult with an ethics committee to ensure data are shared in accordance with participant consent and all applicable local laws. Data sharing should never compromise participant privacy. It is therefore not appropriate to publicly share personally identifiable data on human research participants. The following are examples of data that should not be shared:

-Name, initials, physical address
-Ages more specific than whole numbers
-Internet protocol (IP) address
-Specific dates (birth dates, death dates, examination dates, etc.)
-Contact information such as phone number or email address
-Location data
-ID numbers that seem specific (long numbers, include initials, titled “Hospital ID”) rather than random (small numbers in numerical order)

Data that are not directly identifying may also be inappropriate to share, as in combination they can become identifying. For example, data collected from a small group of participants, vulnerable populations, or private groups should not be shared if they involve indirect identifiers (such as sex, ethnicity, location, etc.) that may risk the identification of study participants.

Additional guidance on preparing raw data for publication can be found in our Data Policy (<https://journals.plos.org/plosone/s/data-availability#loc-human-research-participant-data-and-other-sensitive-data>) and in the following article: <http://www.bmj.com/content/340/bmj.c181.long>.

Please remove or anonymize all personal information (<specific identifying information in file to be removed>), ensure that the data shared are in accordance with participant consent, and re-upload a fully anonymized data set. Please note that spreadsheet columns with personal information must be removed and not hidden as all hidden columns will appear in the published file.

Response: These are elephants, not human patients and the identifying information are relevant to the viral dynamics within the herd. Approval to include this demographic data was obtained from the veterinary departments responsible for the care of the elephants. If the demographic data is still in breach of the PLoS ONE data policy, I can remove the demographic table, however I believe it holds relevant information to the reader.

11. If the reviewer comments include a recommendation to cite specific previously published works, please review and evaluate these publications to determine whether they are relevant and should be cited. There is no requirement to cite these works unless the editor has indicated otherwise.

Response: One additional study was recommended to cite, and has been included due to its use in drawing comparisons in viral loads and clinical progression (reference [62]). A second additional reference has been added to reference treatment protocols used [58].

12. Please review your reference list to ensure that it is complete and correct. If you have cited papers that have been retracted, please include the rationale for doing so in the manuscript text, or remove these references and replace them with relevant current references. Any changes to the reference list should be mentioned in the rebuttal letter that accompanies your revised manuscript. If you need to cite a retracted article, indicate the article’s retracted status in the References list and also include a citation and full reference for the retraction notice.

Response: No other changes to the reference list were required, nor were any references to papers that have been retracted.

**Reviewer 1:**

The manuscript by Wheelahan et al describes a lethal hemorrhagic disease case caused by EEHV1B in an elephant that resided in an Australian zoo. In addition to clinical findings, a complete genome sequence of EEHV1B was determined from heart tissue of the deceased animal. Few cases of EEHV1B have been described and correspondingly, there is little information available for the genomic landscape of EEHV1B strains or genotypes. Overall, this is a well written, comprehensive description of a rare case of EEHV1B-associated hemorrhagic disease and addition of another 1B genome sequence is an important contribution. While there is detailed discussion of finer aspects and peculiarities of the genome sequence, much of the speculation is not inherently novel above many of the observations put forth by several publications from the Hayward lab group.

Thank you for taking the time to review this report. We appreciate the fair and succinct review.

Minor points:
1. page 24, line 516 “ age is an unreliable indicator”. This sentence is somewhat misleading and should modified. While the elephant in this case was just out of the normal range of observed EEHV HD cases, the age range 2-8 is still a predominant feature of EEHV HD. In addition, some discussion should be added as to why some elephants could be outliers from this age range.

Response: We concur that age is still a reasonable indicator of risk, in the absence of serology data which provides a more individualised risk profile. The manuscript has been reworded to reflect this. Additionally, we have added further discussion about why elephants may be outliers as listed below:

**L562**: “… age alone may not reflect individual risk of EEHV-HD”

**L570**: “This correlation between age and antibodies is likely due to EEHV exposure risk accruing with age and cycles of lytic infection due to reactivation [61]. Disease occurrence in older elephants is likely caused by delayed novel exposure to a species of EEHV, which may occur due to prolonged periods of latency in herd-mates, small herd sizes resulting in less potential for exposure, or older contact with new elephants latently infected with novel EEHVs [13, 14, 26].

2. page 25, lines 552-553, “EEHV1B shedding in trunk wash samples before and after a clinical case of EEHV1B disease has not been described previously…..”

a. EEHV1B trunk wash shedding has been described before the current study in Fuery et al, 2016, JZWM (Clinical Infection of Two Captive Asian Elephants (Elephas Maximus) With Elephant Endotheliotropic Herpesvirus 1b).

b. In fact, the authors should consider comparing observations from that study to the current one in either the intro or discussion or both.

Response: Thank you for the recommended additional reference. We acknowledge that this is a useful study to draw parallels in EEHV1B viral dynamics from and have added additional detail in the discussion to reflect this (New citation [62]). We compare viral dynamics and clinical detail to these cases, as well as exploring possible causes for differences in clinical outcomes (**L587-596**). We agree that this represents documentation of EEHV1B trunk wash viral dynamics, but importantly the prior study examined trunk wash shedding in only the affected individuals and not across the broader herd following the viraemias (**L620-623**).

**Reviewer 2:**

The manuscript provides valuable information on the first reported case of EEHV1B infection in zoo Asian elephants in Australia, with important genetic insights that will interest the readership. However, several points require clarification or expansion to strengthen the manuscript. Please address the following comments to improve clarity, accuracy, and completeness.

Thank you for taking the time to review this report. We appreciate the fair and thorough review.

**Title:** As the manuscript primarily focuses on genetic characterization, I encourage

revising the title to more clearly reflect the genetic or whole-genome analysis aspect

of this first EEHV1B case in Australia.

Response: Thank you for the suggestion to revise the title. We have now reworded as follows:

“Virological investigation and comparative genomic analysis of elephant endotheliotropic herpesvirus 1B infection in an Australian captive herd of Asian elephants (*Elephas maximus*)”

**General Comments:** The manuscript inconsistently uses multiple identifiers for the

same individual/sample (e.g., *Elephant 7*, *AUP_01*, *EEHV1B_AUP_01_2023*).

Please standardize terminology throughout to avoid confusion.

Response: To clarify, Elephant 7 refers to the elephant affected by EEHV-HD, whereas EEHV1B_AUP_01_2023 refers to the EEHV1B genome derived from whole genome sequencing on heart tissue of this elephant, and AUP_01 is an abbreviated identifier for in-text brevity.

The nomenclature used in this paper is as follows: within the abstract and at the first introduction of the genome the entire identifier is used, whereas any in-text reference (including in subheadings, figure captions) uses the abbreviated AUP_01. Figures have been updated to use AUP_01, and for the SplitsTree figures which compare to other EEHV sequences, the figures have been updated to conform with the other sequence labels ([Genbank accession #] AUP_01 EEHV1B).

The in-text clarification is found on **L254** of the manuscript: “The full genome sequence of EEHV1B_AUP_01_2023 (herein referred to as AUP_01) …”.

**Materials and Methods**

***Line 152 – Sample collection***

Please clarify the sampling frequency of trunk washes. Were samples collected

every two weeks, monthly, or only opportunistically? The current description is

unclear.

Response: Samples were scheduled to be collected fortnightly however in some instances were not collected due to lack of elephant compliance or time availability. We have corrected the Methods (**L152**) to reflect this: “Trunk wash samples from herd members were collected fortnightly for assessment of EEHV shedding…”.

We similarly updated the whole blood sampling information to provide further context for the collection of these samples: (**L167**): “Whole blood samples were collected from elephants in the herd weekly until eight years of age, fortnightly until nine years of age and monthly from nine years of age, or at any sign of illness.”

***Line 172 – Preservation methods***

Because the study compares different preservation types, please provide more detail

on the storage protocol, including:

• Approximate tissue size or weight placed in each preservative

• Volume (mL) of preservative used

This information is important for interpreting differences between preservation

methods.

Response: Additional sample collection and storage information has now been added as follows:

**L175**: “… (approximately 1g of each tissue) …”

**L180**: “Approximately 3mL of each preservative solution were added to each tissue sample…”

***Line 196 – qPCR platforms***

Different qPCR machines were used for tissue versus trunk wash/whole blood

samples. Please explain the rationale and would this effect the interpretation?

Response: Tissue samples and trunk wash samples were analysed in different laboratories (on-site laboratory testing of trunk washes, University laboratory testing of tissue samples), hence different qPCR platforms were performed. Standard curve characteristics (using known quantities of target region) across the two platforms were comparable. Notably, results between the two platform are not needed to be directly compared, as one was used for analysis of tissue viral loads, whereas the other was used for clinical sample viral surveillance. A clarifying statement has been added:

**L197**: “Different qPCR platforms were used to test tissue samples compared with the whole blood and trunk wash samples due to differences in available instruments across laboratories where the samples were tested.”

**Results**

***Lines 321–336 – Clinical progression***

• Please include the treatment protocol and platelet levels during Days 0–3, as

these provide essential context for interpreting clinical progression alongside

viral load dynamics.

Response:

Thank you for the suggestion. We acknowledge that including the treatment protocols and changes in haematology values would provide valuable context for interpreting the clinical progression in conjunction with the viral loads. However, this paper is focused on the virological investigation and characterisation, rather than a detailed clinical description.

The detailed data relating to clinical treatment and responses belong to the associated veterinary departments at Zoos Victoria and unfortunately, are not available for use in this study. We have, however, added a statement clarifying that treatment was guided by the protocols published by the EEHV Advisory group **(L345)**. The owners of the clinical data intend to publish this information separately, and we anticipate that this report will provide the clinical detail requested.

While we agree that additional clinical detail would make for a more complete case description, we are confident the conclusions of the paper are well supported by the presented virological data.

• Line 329 reports *100,000 VGE/mL* on Day 0, whereas Line 334

reports *4,210,000 VGE/mL*. Please verify which value is correct.

Response: The two different values were determined in different laboratories- the initial 100,000 VGE/mL was an estimated viral load based on Cq value alone, without accurate quantification by real-time comparison of fluorescence to a standard curve (external laboratory service).

The subsequent calculated viral loads (**L350**) were determined by the authors at a separate laboratory, and viral loads were calculated by comparing real-time fluorescence to standard curves of known quantities of virus, utilising the qPCR quantification software.

To add clarification to the report, the first value is now reported as an estimated viral load of greater than 100,000 VGE/mL (**L343**), as well as the rising viraemia clarified as >1,000,000 VGE/mL at day 2 (**L347**).

***Lines 337–364 – Trunk wash results***

It is unclear whether trunk wash positives represent EEHV1B specifically or EEHV1

more generally. For example, Line 343 refers to an EEHV1B spike in Elephant 2, but

Figure 1 labels results as EEHV1. Please clarify:

• Which results distinguish EEHV1A vs EEHV1B

• Which samples were typed as EEHV1B

• Ensure consistent terminology in both text and figures.

Response: Thanks for identifying the inconsistency of reporting this throughout the report. All EEHV1s detected in this study were subtyped to EEHV1B and no EEHV1A was detected. However, viral load quantification was determined from the EEHV1 assay and standard curve (not the EEHV1B subtyping assay), hence the discrepancy between the terms. To improve clarity, additional information has been added:

**Methods** - **L216**: “Quantification of EEHV1 positives was determined from the EEHV1 assay rather than the EEHV1A/B subtype qPCR assays.”.

**Results**: Each reference to EEHV1 positive in the Results (**L359-369**) are now listed as EEHV1B positives. The previous qualifying line (**L372**) “Each of these EEHV1 positives were subsequently subtyped as EEHV1B” which is now superfluous, has subsequently been removed.

**L377:** “No EEHV1A was detected within the study period”

**L386, L423** Updated Fig 1 and Fig 2 labels

***Figure 1***

The x-axis labels are not clear. Please revise to improve readability and

interpretation.

Response: We have updated Fig. 1 to have clearer x-axis labels (one tick and label per study month), and have updated the caption to identify the reason for the x-axis break (sampling not undertaken) in the Figure caption (**L391**).

**Discussion**

In addition to including treatment details in the Results, please expand the

Discussion to address treatment outcome, including factors contributing to treatment

success or failure.

Response:

As outlined above, the detailed data relating to clinical treatment and responses belong to the associated veterinary departments at Zoos Victoria and are not available for use in this study. The focus of this report is a virological investigation, though we acknowledge that there is some limitation to assessing viral loads in absence of clinical data. We have added some further discussion by comparing the case to two surviving EEHV1B cases based on the available data, as suggested by Reviewer 1 (**L569-578**).

**Additional point regarding preservation performance:**

The manuscript does not discuss why RNAlater appeared ineffective for most tissue

samples. Please expand on potential explanations.

Additionally, for samples preserved under different conditions—including non-

preserved tissues—please discuss why some yielded “N/A” results. In theory, non-

preserved samples should still contain detectable host DNA. Please consider

whether these outcomes may reflect:

• Variation in DNA extraction quality

• Tissue degradation

• Differences in preservation performance

• Personal error

A brief discussion of these possibilities will help readers interpret the preservation

comparison.

Response: N/A results indicate that the tissue was not stored in that particular medium (when collected by the pathologist) and thus not available for testing (as identified in the table footnotes [**L421**]), rather than lack of detection of viral or elephant DNA. Figure 2 shows that RNA later was equivocal to other storage media for recovery of viral and host DNA for the four tissues in which it was available.
